# Brain state identification and neuromodulation to promote recovery of consciousness

**DOI:** 10.1093/braincomms/fcae362

**Published:** 2024-10-11

**Authors:** Glenn J M van der Lande, Diana Casas-Torremocha, Arnau Manasanch, Leonardo Dalla Porta, Olivia Gosseries, Naji Alnagger, Alice Barra, Jorge F Mejías, Rajanikant Panda, Fabio Riefolo, Aurore Thibaut, Vincent Bonhomme, Bertrand Thirion, Francisco Clasca, Pau Gorostiza, Maria V Sanchez-Vives, Gustavo Deco, Steven Laureys, Gorka Zamora-López, Jitka Annen

**Affiliations:** GIGA-Consciousness, Coma Science Group, University of Liège, Liège 4000, Belgium; Centre du Cerveau, University Hospital of Liège, Liège 4000, Belgium; Institut d'Investigacions Biomèdiques August Pi i Sunyer (IDIBAPS), Clinical and Experimental Neuroscience, Barcelona 08036, Spain; Institut d'Investigacions Biomèdiques August Pi i Sunyer (IDIBAPS), Clinical and Experimental Neuroscience, Barcelona 08036, Spain; Institut d'Investigacions Biomèdiques August Pi i Sunyer (IDIBAPS), Clinical and Experimental Neuroscience, Barcelona 08036, Spain; GIGA-Consciousness, Coma Science Group, University of Liège, Liège 4000, Belgium; Centre du Cerveau, University Hospital of Liège, Liège 4000, Belgium; GIGA-Consciousness, Coma Science Group, University of Liège, Liège 4000, Belgium; Centre du Cerveau, University Hospital of Liège, Liège 4000, Belgium; GIGA-Consciousness, Coma Science Group, University of Liège, Liège 4000, Belgium; Centre du Cerveau, University Hospital of Liège, Liège 4000, Belgium; Swammerdam Institute for Life Sciences, University of Amsterdam, Amsterdam 1098XH, Netherlands; GIGA-Consciousness, Coma Science Group, University of Liège, Liège 4000, Belgium; Centre du Cerveau, University Hospital of Liège, Liège 4000, Belgium; Barcelona Institute of Science and Technology, Institute for Bioengineering of Catalonia (IBEC), Barcelona 08028, Spain; Biomedical Research Networking Center in Bioengineering, Biomaterials, and Nanomedicine (CIBER-BBN), Madrid 28029, Spain; GIGA-Consciousness, Coma Science Group, University of Liège, Liège 4000, Belgium; Centre du Cerveau, University Hospital of Liège, Liège 4000, Belgium; Department of Anesthesia and Intensive Care Medicine, Liege University Hospital, Liege 4000, Belgium; Anesthesia and Perioperative Neuroscience Laboratory, GIGA-Consciousness Thematic Unit, University of Liege, Liege 4000, Belgium; Inria, CEA, Université Paris-Saclay, Paris 91120, France; Department of Anatomy and Neuroscience, School of Medicine, Universidad Autónoma de Madrid, Madrid 28029, Spain; Barcelona Institute of Science and Technology, Institute for Bioengineering of Catalonia (IBEC), Barcelona 08028, Spain; Biomedical Research Networking Center in Bioengineering, Biomaterials, and Nanomedicine (CIBER-BBN), Madrid 28029, Spain; Institució Catalana de Recerca i Estudis Avançats (ICREA), Barcelona 08010, Spain; Institut d'Investigacions Biomèdiques August Pi i Sunyer (IDIBAPS), Clinical and Experimental Neuroscience, Barcelona 08036, Spain; Institució Catalana de Recerca i Estudis Avançats (ICREA), Barcelona 08010, Spain; Institució Catalana de Recerca i Estudis Avançats (ICREA), Barcelona 08010, Spain; Computational Neuroscience Group, Center for Brain and Cognition, Universitat Pompeu Fabra, Barcelona 08018, Spain; GIGA-Consciousness, Coma Science Group, University of Liège, Liège 4000, Belgium; Centre du Cerveau, University Hospital of Liège, Liège 4000, Belgium; Joint International Research Unit on Consciousness, CERVO Brain Research Centre, University of Laval, Quebec G1J 2G3, Canada; International Consciousness Science Institute, Hangzhou Normal University, Hangzhou 311121, China; Computational Neuroscience Group, Center for Brain and Cognition, Universitat Pompeu Fabra, Barcelona 08018, Spain; GIGA-Consciousness, Coma Science Group, University of Liège, Liège 4000, Belgium; Centre du Cerveau, University Hospital of Liège, Liège 4000, Belgium; Department of Data Analysis, University of Ghent, Ghent B9000, Belgium

**Keywords:** brain states, neuromodulation, (disorders of) consciousness, anaesthesia, animal models

## Abstract

Experimental and clinical studies of consciousness identify brain states (i.e. quasi-stable functional cerebral organization) in a non-systematic manner and largely independent of the research into brain state modulation. In this narrative review, we synthesize advances in the identification of brain states associated with consciousness in animal models and physiological (sleep), pharmacological (anaesthesia) and pathological (disorders of consciousness) states of altered consciousness in humans. We show that in reduced consciousness the frequencies in which the brain operates are slowed down and that the pattern of functional communication is sparser, less efficient, and less complex. The results also highlight damaged resting-state networks, in particular the default mode network, decreased connectivity in long-range connections and especially in the thalamocortical loops. Next, we show that therapeutic approaches to treat disorders of consciousness, through pharmacology (e.g. amantadine, zolpidem), and (non-) invasive brain stimulation (e.g. transcranial direct current stimulation, deep brain stimulation) have shown partial effectiveness in promoting consciousness recovery. Although some features of conscious brain states may improve in response to neuromodulation, targeting often remains non-specific and does not always lead to (behavioural) improvements. The fields of brain state identification and neuromodulation of brain states in relation to consciousness are showing fascinating developments that, when integrated, might propel the development of new and better-targeted techniques for disorders of consciousness. We here propose a therapeutic framework for the identification and modulation of brain states to facilitate the interaction between the two fields. We propose that brain states should be identified in a predictive setting, followed by theoretical and empirical testing (i.e. in animal models, under anaesthesia and in patients with a disorder of consciousness) of neuromodulation techniques to promote consciousness in line with such predictions. This framework further helps to identify where challenges and opportunities lay for the maturation of brain state research in the context of states of consciousness. It will become apparent that one angle of opportunity is provided through the addition of computational modelling. Finally, it aids in recognizing possibilities and obstacles for the clinical translation of these diagnostic techniques and neuromodulation treatment options across both the multimodal and multi-species approaches outlined throughout the review.

## Graphical Abstract

**Graphical abstract:**
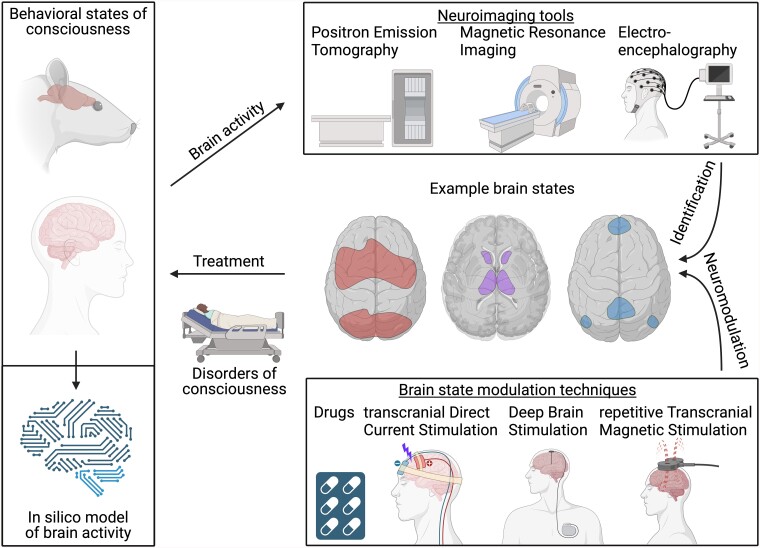

## Teaser

Van der Lande *et al.* build a framework to integrate modes of brain activity (i.e. brain states) and their association with (un)consciousness. Slower and less complex brain states are associated with unconsciousness. The framework can then be utilized to inform and improve clinical and preclinical neuromodulation techniques to promote more complex brain states to potentially treat disorders of consciousness.

## Introduction

Consciousness is the foundation of human experience, it refers to a person's awareness of something—‘What it is like’.^1^ Although universal agreement on the brain mechanisms supporting consciousness remains elusive, this does not prevent the development of fundamental and clinically useful knowledge. The investigation of brain states, spatiotemporal patterns of neuronal activity (see Box 1) and their dynamics across states of consciousness alongside their coupling to behaviour can help advance the field.

Box 1 **–What are brain states?**

A brain state is a temporary configuration of brain activity. It is a quasi-stable state with minimal fluctuation within the state and large fluctuations between other states. Brain states are objective, as each corresponds to a specific pattern of electrical or chemical activity, that can be measured using various neuroimaging and neurophysiological techniques. They are parallel to states of consciousness, or mental states, which represent the arousal, awareness and content of consciousness of an individual at a given time, which we refer to as subjective experience. This can range from, amongst others, conscious wakefulness to meditation, drug-induced states, sleep, anaesthesia and coma. All mental states have one or multiple associated brain state(s), and different dynamics seem important to distinguish between states of e.g. consciousness and unconsciousness. However, with the brain operating on multiple spatial (e.g. single neurones to the whole brain) and temporal (e.g. action potentials to persistent functional connectivity networks) scales, it is impossible to capture the entire brain state for every mental state. As a result, approaches for the quantification of brain states differ in spatial and temporal scale, yielding siloed lines of inquiry and subfield-specific definitions. Moreover, investigation of the brain often resorts to proxies of neural activity (e.g. blood-oxygen level-dependent (BOLD) signals). Therefore, the investigation of brain states relies on capturing vital parts of functional brain configurations to enable specific behaviours (e.g. connectivity to and specific activity within the motor cortex is required to make certain movements) or subjective experiences. It follows that the investigation of brain states is a potentially good proxy for quantifying states of consciousness. The challenge is how to identify and differentiate between those based on empirical observations of the brain's spatiotemporal activity.

Research on the identification of brain states lacks clear definitions of what a brain state precisely is.^2^ Going forward, brain states should quantify and clearly describe which multi-dimensional perspective is the most adapted to consciousness research. Here, we employed a rather broad definition, incorporating a large array of features extracted from the brain. Frequency ranges play a significant role in defining brain states, with slow delta oscillation being mostly associated with unconsciousness, while alpha and, to a lesser extent, theta, beta, and gamma oscillations being related to states of consciousness. Well-organized yet flexible functional communication between brain regions, and the loose coupling of functional connectivity to structural connectivity, is crucial for the emergence of conscious states. The ability to orchestrate complex temporal dynamics, by dynamically crossing a wide range of network configurations to allow for appropriate multisensory integration is paramount for consciousness. Dynamic analysis tools, such as those investigating the complexity of functional connectivity patterns (e.g. the study by Demertzi *et al*.^3^ in Fig. 2C) bring along promising opportunities to not only describe a single state but to explore the importance of their temporal dynamics. The transitions between brain states typically are non-linear, or critical. As a consequence, transitions between states can become scarcer in a damaged brain. Hence, in some cases, there could be evidence of more persistent states, for example, dictated by slow waves,^4^ highlighting the necessity of investigating dynamics. Here, we refer to brain states in general, or to specific features of these brain states when required.

Dynamic brain states form a rich repertoire associated with different states of consciousness, as clearly demonstrated by the diversity of brain states in sleep. Brain state dynamics typically do not follow linear transitions, but are changing in a critical manner. Brain states associated with states of (un)consciousness are usually regarded as whole brain, with specific spatiotemporal dynamics. We here focus on literature that has investigated global brain states associated with (un)consciousness to give an overview of key elements of brain function that support normal consciousness.

In the clinical domain, consciousness is often divided into an arousal component and an awareness component, which respectively refer to wakefulness (eye-opening) and the subjective experience one can have.^7^ The full neural correlate of consciousness (NCC) is the brain state that supports these dimensions while a specific NCC supports specific conscious content.^8^ The collection of all specific NCCs (e.g. motion perception), mostly located in the temporo-parietal-occipital hot zone, could be considered as the building ground for the full NCC as well.^9^ Facilitating conditions, such as activity in the ascending arousal network (AAN; also referred to as ascending reticular activating system) and the thalamus,^10^ that shape brain states can be identified. Indeed, functional magnetic resonance imaging (fMRI) and electrophysiology studies suggest that consciousness depends on both large-scale thalamocortical and corticocortical interactions (e.g.^11,12^). These structures, their connections and their outputs shape brain states and their dynamics, as captured, for instance, by whole-brain functional connectivity.^13^

**Figure 1:**
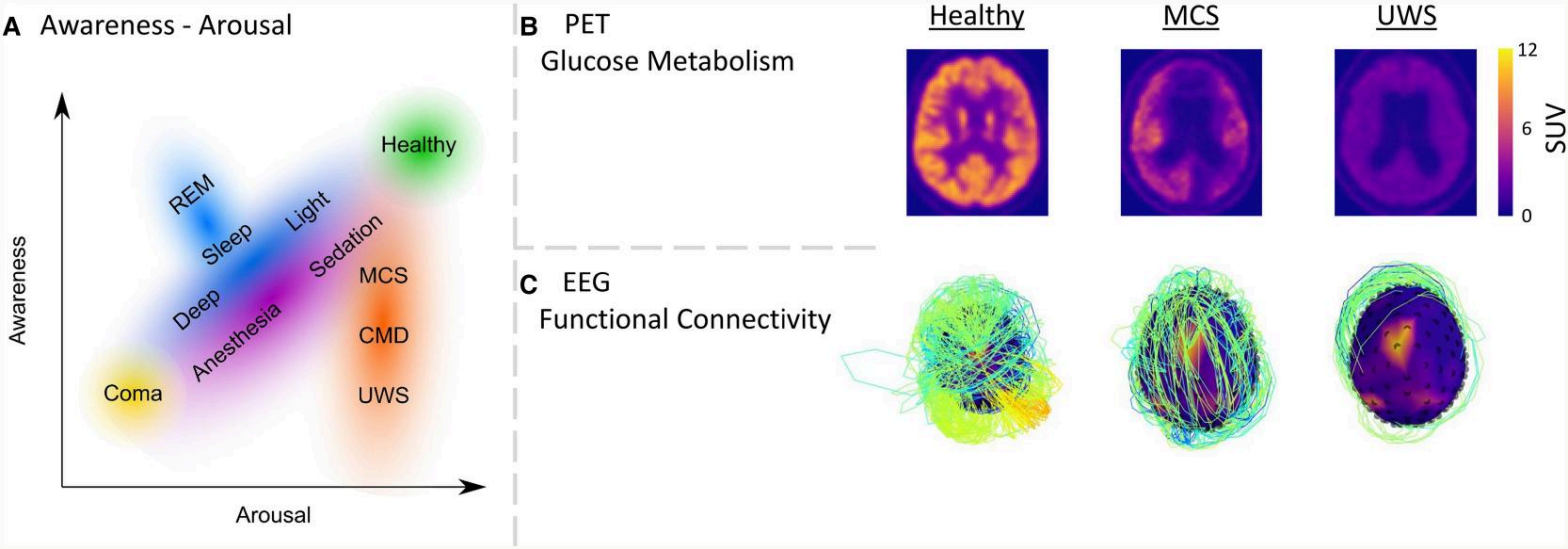
**The arousal-awareness axes of consciousness and examples of metabolic and functional connectivity in patients with disorders of consciousness.** (**A**) Graphical depiction of states of consciousness alongside the dimensions of arousal and awareness including healthy awake consciousness, different states of sleep (light, deep, REM), a scale from sedation to anaesthesia, and the DoC states of the minimally conscious state (MCS), the unresponsive wakefulness syndrome (UWS) and Cognitive Motor Dissociation (CMD). Note that these states exist on continuous scales. (**B**) Transversal view of the standardized uptake value of the brain collected with glucose positron emission tomography (fluorodeoxyglucose/FDG-PET) (see the study by Thibaut *et al*.^5^ for details about data processing). The glucose uptake values range from 1 to 12 where higher values are associated with more glucose consumption, as observed in a healthy brain. (**C**) Scalp mesh with electroencephalogram (EEG) electrode locations indicated as black dots. Lines between electrodes depict the connectivity between functionally connected electrodes. Functional connectivity was determined by the weighted phase lag index^6^ on preprocessed data (see the study by Thibaut *et al*.^5^ for details about the pre-processing). For clarity, the top 5 strongest outgoing connections per electrode are shown. Strength of the connection is represented by the height of the line (i.e. weak connections are low and yellow, and strong connections are high and red). Access to interactive figures that allow for deeper investigation into the concepts illustrated here can be found in the Supplementary material.

Consciousness can be lost during sleep, but also in awake conditions, by a disconnection from the outside world and the loss of the sense of self. Prolonged loss of consciousness can happen after severe brain injury leading to a coma, as presented in patients with a disorder of consciousness (DoC; Fig. 1).^14^ These patients show eye-opening after coma, but do not recover (full) awareness. Several types of brain injury can lead to a DoC, including traumatic brain injury, cardiac arrest, haemorrhage or infection.^15^ Prolonged DoC is considered a rare disease, affecting between 0.2 and 17 individuals per 100.000 in Europe and the United States.^16-23^ Behaviourally, patients with a DoC can be further split into those with a complete absence of awareness, i.e. patients with the unresponsive wakefulness syndrome (UWS^24^) or vegetative state or those with partially preserved awareness, i.e. patients in the minimally conscious state (MCS^25^; Fig. 1A). Especially, this latter group represents a very heterogenous population, including patients who only present limited signs of awareness, such as visual pursuit (MCS-^26^) and patients who show language-dependent signs of awareness, such as command following (MCS + ^26^). Other patients are unable to show behavioural signs of awareness but yet have relatively preserved neural function at rest (MCS*^5^), during passive stimulation (higher order motor dissociation or covert cortical processing^27,28^), or active tasks (cognitive motor dissociation (CMD)^29^; see the study by Meys *et al*.^30^ for an overview). After the brain injury, patients can transition between these states both in the acute phase (<28 days from onset), in the prolonged stage thereafter (>28 days), up to years after the injury,^31^ or might never recover from the DoC. Patients recovering from MCS within 3 months (for anoxia) or 1 year (for TBI) have higher chances to recover consciousness than those remaining in UWS for an extended period of time.^32^ Consciousness is usually assessed behaviourally, typically with the Coma Recovery Scale-Revised (CRS-R^33^). Misdiagnosis based on behavioural examination of patients with a DoC is common, especially when not performed repeatedly.^34^ Being able to identify the state of a patient with precision is thus crucial to safeguard against misdiagnosis. This is where precise and reliable methods for brain state identification have been recognized.^35^ For example, capturing the metabolic brain state of patients with a DoC may safeguard against misdiagnosis, as seen when brain states similar to those in MCS patients are identified in UWS patients, suggesting that the patient may have (partially) preserved awareness.^5^

While research with the DoC population has given new insights into the fundamentals of consciousness and improvement of care for those suffering from DoC, clinical and ethical constraints limit the empirical possibilities. Promoting recovery of consciousness is paramount in future research for both fundamental reasons and as a tool for clinical improvements. Behaviourally, pathological (e.g. DoC), physiological (e.g. sleep) and pharmacological (i.e. sedation and anaesthesia) states of unconsciousness may have a strong resemblance, but it is unsure how much the associated brain states are equal.^36-39^

Neuromodulation techniques (Box 2) can be used to alter brain states and maybe even transition between them. For example, to promote consciousness and as such potentially serve as a curative treatment for patients with a DoC. For example, transcranial direct current stimulation (tDCS) can reduce slow-wave activity usually associated with the absence of consciousness.^4^ There is a variety of non-invasive approaches to modulate neuronal activity including magnetic and electrical stimulation, ultrasound and near-infra-red laser light,^40^ as well as invasive methods such as deep brain stimulation (DBS), that have shown potential to induce brain state changes in both human and animal models.^41^ Indeed, animal studies offer excellent experimental control and the behavioural effects of induced brain-state changes can be assessed through the detection of increased movements and normalized vital signs, for instance.^42^ Although the heterogeneity of brain injuries in patients with a DoC makes it challenging to create generalizable relevant animal models,^43^ they constitute important advances. In a recent study, coma was induced in rats by lesioning the tegmentum of the brain stem, and their recovery was described in terms of reactivation of thalamocortical functional connectivity.^44^

Box 2 **—Neuromodulation**

With neuromodulation, we here refer to any exogenous intervention that is applied to change neuronal activity. Neuromodulation can serve to define a new line of treatments by inducing brain state transitions with the potential to promote consciousness. Neuromodulation can be invasive or non-invasive, and neuronal processes can be targeted using various techniques. They can be grouped into two categories: chemical and electromagnetic physical stimulation. Chemical alterations can be made through pharmacological interventions, for instance targeting the ion channels that increase or decrease neurones’ likelihood of producing action potentials. Electrical or magnetic stimulation can change neuronal behaviour by inducing an extracellular flow of current, and an artificial neuronal hyper- or depolarization. Radiofrequency, ultrasound, or infra-red neural stimulation can also be used for neuromodulation, although they may not yet be considered conventional techniques. Neuromodulation techniques have already demonstrated their efficacy in other clinical areas, for instance with the use of transcranial direct current stimulation (tDCS) to treat central sensitization syndromes (fibromyalgia) and depression,^45^ and the use of deep brain stimulation (DBS) for Parkinson's disease.^46^ While neuromodulation is considered a safe and effective treatment for a multitude of diseases, its equivalent potential for the treatment of DoC is still far from optimal.

The ability to promote recovery of consciousness in patients with a DoC will flow from two main lines of research. First, identifying how brain states differ between consciousness and unconsciousness, and between pathological and healthy conditions. Second, developing theoretical and empirical tests to induce transitions between different states of consciousness, or from pathological to healthy states. Here, we introduce a model that integrates the two distinct disciplines of brain state identification and modulation. With this, we aim to forge a framework where their synergy facilitates progress towards better clinical care and furthering the understanding of brain dynamics and how they underlie consciousness. Then, we will describe the known relationships between consciousness and brain states. We will briefly review the evidence for the importance of investigating brain states during loss and recovery of consciousness. The next section discusses the induction of state changes through potential curative treatments for DoC, where we discuss four options including pharmacology, photopharmacology, non-invasive brain stimulation, and finally deep brain stimulation. A comprehensive discussion highlights the limitations of the current state-of-the-art and potential future perspectives.

### Therapeutic framework for identification and modulation of brain states

**Figure 2:**
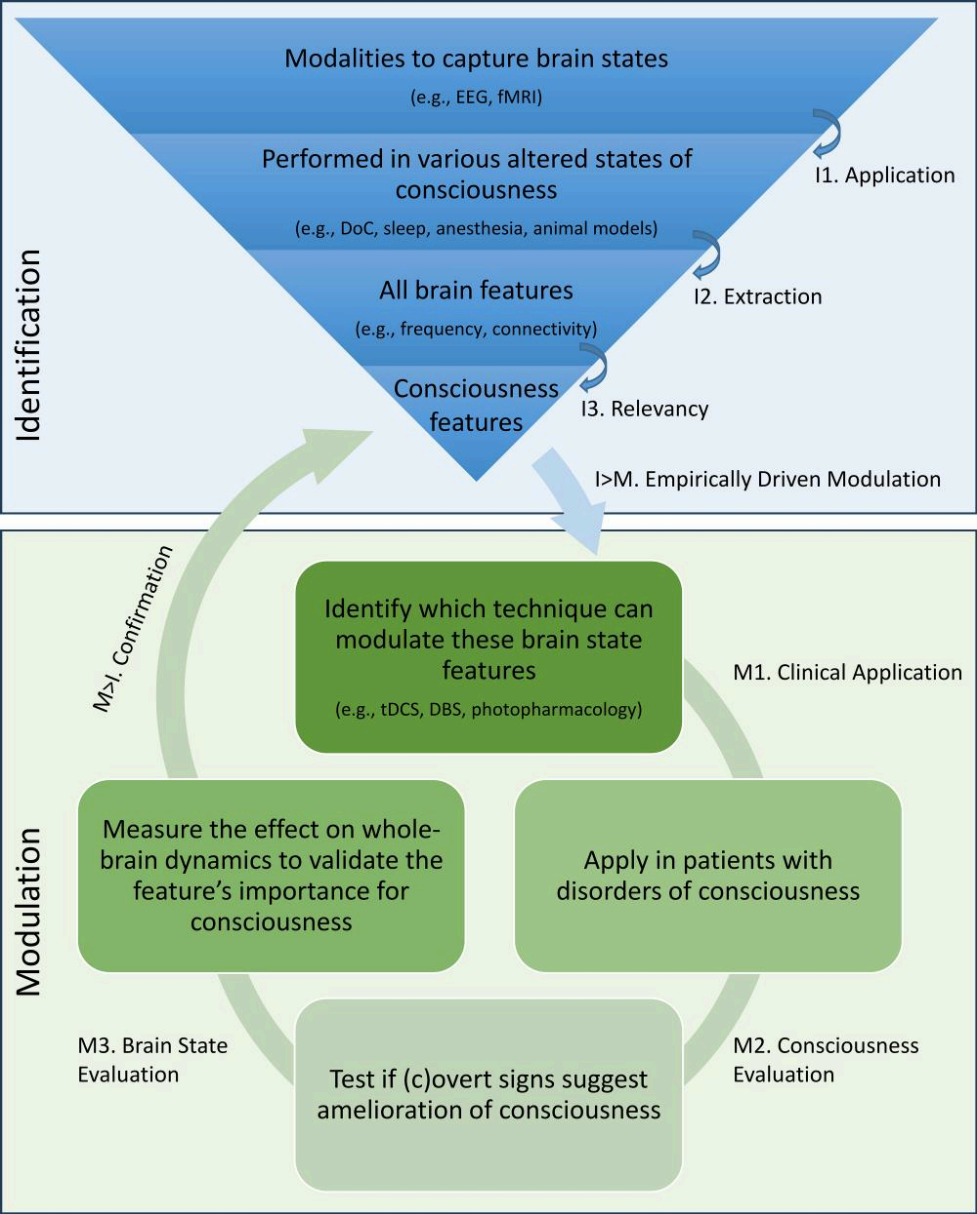
**Therapeutic framework for identification and modulation of brain states.** The framework consists of two parts: Identification and Modulation part that interact and support each other. Within each part, several levels of approaches can be distinguished, all of which are important for brain state research. From the top, the research focusing on brain state identification can utilize and develop techniques, such as electroencephalography (EEG) and magnetic resonance imaging (MRI), to capture the brain state, which can then be applied in various altered states of consciousness, such as disorders of consciousness (DoC) (*I1*), from which features can be extracted (*I2*), that can be evaluated on the relevancy for consciousness (*I3*). In the bottom part, modulation techniques, such as transcranial direct current stimulation (tDCS) and deep brain stimulation (DBS), can be identified to modulate these features, which could then be applied in patient populations (*M1*), where alterations in (c)overt signs of consciousness could be evaluated (*M2*), followed by an investigation on the whole-brain dynamics to examine brain state changes (*M3*). These two sides of the field are linked where successful manipulation of a feature, associated with amelioration of consciousness, could confirm its relevance as a feature of consciousness (*M > I*), and the other way around, relevant features could drive the direction of the neuromodulation field empirically (*I > M*). This framework is flexible enough to accommodate a variety of approaches and data types, but also provides enough structure to ensure consistency and rigour spanning from single unit recordings to whole-brain recordings in different levels of consciousness. It is adaptable to new discoveries and technologies as they emerge in various fields.

To achieve better clinical care for patients with a DoC, from diagnosis to treatment, the identification and modulation of brain states should encompass a comprehensive integrated view. This field addresses several central research lines, including (1) the development of new algorithms to analyse brain activity and associate resulting features with different states of consciousness, (2) design interventions to induce specific brain states associated with consciousness, (3) evaluate the effectiveness of different brain state manipulation techniques in ameliorating consciousness, and (4) understanding the underlying mechanisms of brain state dynamics. We propose an approach that ties these facets together, as these seemingly independent questions are intertwined and have the potential to inform each other with insights from different disciplines, including but not limited to animal and human neuroscience, psychology, physics and computer science. The flexible framework (Fig. 2) provides a set of principles and guidelines for developing and applying techniques to both measure and manipulate brain states.

The study of brain function associated with consciousness is not limited to a specific modality and can span different temporal resolutions (i.e. from milliseconds to minutes), spatial resolutions (e.g. single unit recordings, neural populations or networks) and can quantify neural activity directly (e.g. action potentials) or proxies thereof (e.g. glucose uptake). Each technique has its own strength and unique contribution. Hence, within the framework, no restrictions are placed on the modality for the assessment of consciousness. through for instance, electroencephalogram (EEG), fMRI, positron emission tomography (PET), and electrode recordings. These techniques should be applied to identify brain states (*I1*) in pathological altered states of consciousness, as a result of brain injury, the physiologically altered states of sleep, meditation or hypnosis, and the pharmacologically altered states of anaesthetic or psychedelic drugs in human participants, animal analogues or in silico. Data from all these angles would allow the extraction (*I2*) of features of brain states potentially related to consciousness, which could be extracted (*I3*) with appropriate statistical or machine-learning procedures. This would allow for a universal characterization of brain states related to consciousness and the development of bottom-up, empirically-inspired (*I > M*) and targeted neuromodulation techniques.

While currently several treatments for patients with a DoC are being tested and validated, they are usually based on a specific target location or circuitry rather than whole-brain dynamics. Stimulation is in the first place location-bound, which might have contributed to the limited treatment efficacy reported up to date. Within the currently limited possibilities for application, neuromodulation techniques targeting relevant network dynamics should be applied in clinical populations (*M*1). The effect on the level of consciousness and behaviour (*M*2) should be systematically assessed, both at the behavioural level and at the brain level. It is essential to measure the effect on whole-brain dynamics and brain states as well (*M*3), to verify if the specific treatment had the desired effect on global dynamics. Together with the evaluation of (c)overt signs of consciousness (*M*2), neuromodulation techniques are a gateway to more causal inferences if specific brain states are truly related to consciousness. If not, the quantification of the effect on the brain and behaviour might allow the refinement of brain states causally involved with consciousness, particularly if more prominent alterations are observed in other aspects of whole-brain dynamics. In this way, the function of brain states could be empirically tested, potentially enabling the confirmation (*M > I*) of the NCC. This framework is flexible enough to accommodate a variety of approaches and data types and also provides enough structure to ensure consistency and rigour.

### Consciousness and brain states

The research on brain states opens the window to a common language and toolboxes aiming at increasing the understanding of the brain.^47^ The development of reliable biomarkers of consciousness, which can be used as diagnostic tools for patients with a DoC has important implications regarding ethical considerations (e.g. end-of-life decisions^48^), pain treatment,^49^ curative treatment,^50^ and prognosis.^51^

### Identification of brain states using electrophysiology

Brain states can be studied with a high level of spatiotemporal details, potentially allowing for a fine-grained assessment of their link with subjective experience. However, also single feature-based classification using electrophysiology has been robustly linked with broad classes of conscious states (*I2*). Prominently, alterations in the amplitude of electrical activity in different frequency bands, delta (1–4 Hz), theta (4–8 Hz), alpha (8–12 Hz), beta (12–30 Hz) and gamma (>30 Hz) have been used in sleep studies for that purpose.^52,53^ According to the classical perspective on these brain state features, they can be broadly associated with behavioural states: lower frequency oscillations (e.g. delta) are associated with unconsciousness (although accumulating evidence now suggests the possibility for the presence of dominant delta oscillations in conscious states^54^), while theta waves have been associated with drowsiness^55^ and daydreaming.^56^ Higher frequencies are more often associated with states of higher consciousness. Alpha waves suggest a state of attention^57^ and visual processing^58^ (or in rare cases during coma,^59^ possibly reflecting suppression of cerebral function^60^). Beta waves are common during normal waking conscious states,^61^ and gamma waves are associated with the performance of cognitive tasks,^62^ promoting communication between distant neural populations.^63^

**Figure 3:**
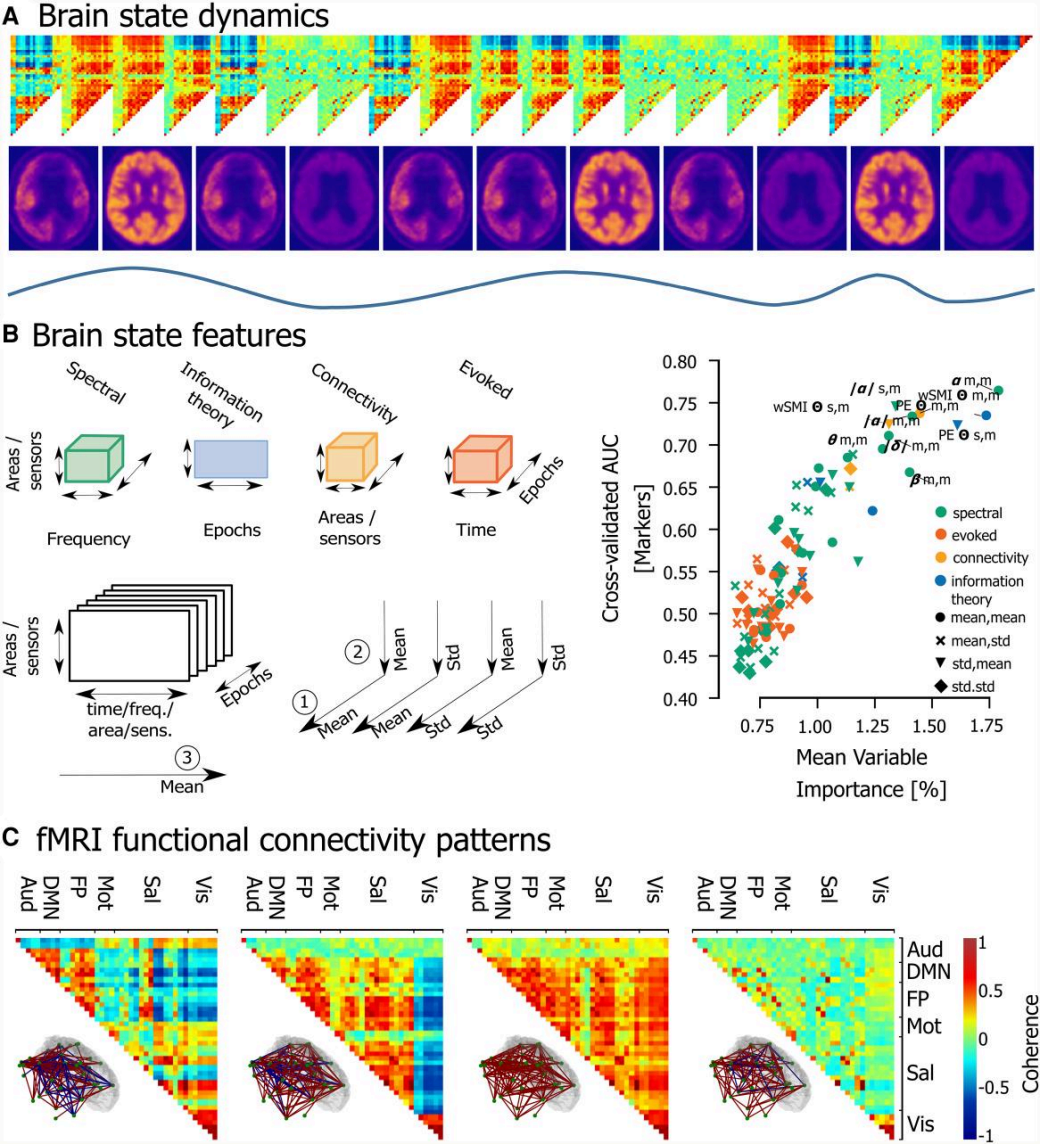
**Methods for identification and characterization of brain state dynamics illustrated through fMRI connectivity patterns. (A)** Illustration of the multi-modal dynamics of brain states showing how they can change with different timescales, while also displaying recurrences in a repertoire. The top shows an illustration of how brain states can be quantified (e.g. from bottom to top: through oscillatory EEG of fMRI activity, glucose metabolism or functional connectivity between regions) and how brain states fluctuate over time. For illustrator purposes, data was taken from 1B and 3C, but illustrated dynamics are purely illustrator and not based on real data. **(B)** The wide range of features that can be used to describe brain states (adapted from the study by Engemann *et al*.^64^). The left figure displays these features in different categories, showing which dimensions they take into account, i.e. samples, time, area, frequency (freq.), sensors (sens.), and various ways to summarize them (e.g. mean, standard deviation (std)). The right figure shows how this was applied to EEG data, with the plentitude of features now ordered based on their importance in predicting if a DoC patient is fully unaware (UWS) or has residual awareness (MCS) (see^64^ for details). This ordering was done based on the cross-validated area under the curve (AUC). This illustrates that multifaceted investigation is important, alongside the need for the selection of the most relevant features for the differentiation between brain states and parallel behavioural state. **(C)** Patterns of functional connectivity that occur in a quasi-stable manner and alternate dynamically could be considered brain states. Functional connectivity is defined between areas in the auditory network (Aud), default mode network (DMN), fronto-parietal network (FP), motor network (Mot), salience network (Sal), and visual network (Vis) defined as 10-mm-diameter spheres around peak coordinates selected from the literature. The top part shows 4 recurring patterns or brain states (for details on their extraction, see the study by Demertzi *et al*.^3^). The bottom shows a representation of the functional connectivity between brain regions for each of these brain states. Positive connections are shown in red and negative ones in blue. The 5% strongest connections are presented. Access to interactive figures that allow for deeper investigation into the concepts illustrated here can be found in the Supplementary material.

The electrophysiological fingerprint of brain states can be challenging to interpret, as these physiological rhythms can be influenced by the presence of pathological activity.^60^ DoC patients display characteristic resting-state EEG features, which can be extracted using mathematical tools that estimate spectral, connectivity, or information theoretical aspects (see Fig. 3B;^64^). By merely visually assessing the power spectrum distribution at specific frequency bands, one can define functional regimes (i.e. A, B, C or D) rooted in the mesocircuit hypothesis of consciousness, which highlights the importance of thalamocortical interactions.^12^ There, the worst functioning category (A) is akin to complete deafferentation, corresponding to an absence of peaks in the power spectrum, while the highest functioning category (D) displays a healthy peak in the theta, alpha and beta ranges.^65^ Progression of patients with an acute DoC along these regimes is predictive of their natural recovery.^66,67^ Others have also shown the relevance of theta, alpha and beta activities. For example, several graph-theory measures of connectivity in the alpha frequency range follow the level of consciousness,^68^ while network centrality in the theta band is associated with a higher probability of a positive response to electrical stimulation.^69^ Indeed, when comparing measures that capture different aspects of the EEG-recorded brain states, power in these frequency ranges, along with the functional connectivity and complexity, appears as a dependable marker of the state of consciousness.^70^ Ameliorating the spatiotemporal definition of brain states might help identify the course of specific activity to characterize brain states more precisely, and to extract (*I2*) the most relevant (*I3*) features for consciousness. It has been shown that reduced integration and increased network segregation characterize patients with a DoC.^71^ EEG microstates also consider the spatial distribution of activity, as they are transient (millisecond to second range), patterned (dipole over the scalp), and quasi-stable (extended periods during which there is small variance) states or patterns over the scalp. They are considered global functional states that function as elementary building blocks of the content of consciousness.^72^ As recently shown, the temporal dynamics of these microstates is predictive of outcomes in patients with a DoC.^73,74^ However, specific behavioural states and subjective experiences cannot be fully described by these coarse spectral classifications.

### Identification of brain states using functional magnetic resonance imaging

Neuroimaging tools provide another way to increase the spatial resolution for the identification of brain states (*I2*). Functional connectivity between brain regions, commonly assessed using fMRI, is an important tool which can identify fluctuating brain states characterized by networks of functional connectivity at rest. These resting-state networks are important for consciousness. For example, the preservation of connectivity in networks that have been associated with ‘internal’ and ‘external’ awareness is associated with the level of consciousness.^75^ Especially the default mode network’s functional connectivity is reduced in patients with a DoC^76,77^ and can be associated with reduced thalamic function.^78^ Other traits of the functional networks of the brain, like the connections in sensory, auditory and motor networks and interhemispheric connectivity, are also reduced.^77,79,80^ More recently, the importance of dynamics within these networks has been emphasized repeatedly. Indeed, the complexity of patterns of functional connectivity has been observed to decrease in MCS patients, and to a greater extent in patients with UWS.^3^ (Figure 3). The decrement in these arousal and awareness-supporting dynamics is linked to a global reduction in functional connections, their diversity and recurrent inputs coupled with more homogenous local dynamics.^81^ The intricate link of functional diversity and its interaction with integration in supporting consciousness, whether causal for or as a result of processing information, has been supported by others.^82^ In addition, patterns of global brain communication in DoC are characterized by reduced transitions between states of functional connectivity.^83^ The amount and occupancy of states in the dynamic functional connectivity repertoire, for instance in the default mode network, of patients with a DoC is reduced in UWS compared to MCS patients.^84^

### Anaesthesia as a model for pathological loss and recovery of consciousness

Anaesthesia can be used as a powerful model for loss of consciousness that propels research into its recovery.^85,86^ From mild sedation to full anaesthesia, the effects depend of the type of anaesthetic and dose. Here, the discussion will focus on any anaesthetic in dosages that lead to a perceived loss of consciousness. Their mechanisms of action have been studied in great detail, allowing the generation of hypotheses for loss and recovery of consciousness in DoC.^87-91^ Anaesthesia induces states with a prominence of low-frequency oscillations^92-95^ and a reduction in high-frequency functional connectivity (85–155 Hz).^96^ It has been suggested that during anaesthesia local connectivity increases, whereas global alpha connectivity decreases.^93^ Although not a direct comparison, healthy controls under anaesthesia have shown a reduction in global connectivity, dynamic repertoire, network topological properties (i.e. integration and segregation) and regional heterogeneity which is similar to that seen in patients with a DoC compared with healthy controls.^81,85^ Another study has shown decreased frontal-parietal connectivity, while thalamocortical connectivity remained unchanged.^97^ However, thalamocortical connections and the AAN, which play a key role in the brain state of patients with a DoC, also appear altered after the application of most anaesthetic agents.^91,98-100^ A direct comparison matched these findings with reduced integration and functional diversity in overlapping brain regions of the posterior cingulate cortex and precuneus in both DoC and under anaesthesia compared to healthy wakefulness.^82^ In addition, the occupancy of dynamic connectivity patterns of low complexity, which increases from MCS to UWS, has similar rates in DoC compared to anesthetized volunteers (Fig. 3C for DoC).^3^ Anaesthesia reduces a wide range of brain state properties like corticocortical and thalamocortical connectivity within and between default mode and executive-control networks.^101-104^ There are important commonalities between the observed brain state changes in pathological and pharmacologically altered consciousness (*I2*), with the important difference that the healthy volunteers undergoing anaesthesia can provide subjective reports after recovery. These studies therefore can serve as a benchmark from which can be extrapolated to the DoC population. The effects of anaesthetics are usually studied in healthy volunteers, however, subjects with brain damage with no disorders of consciousness might be more suitable for this purpose.^105^

It should be noted that different pharmacological agents have different mechanisms of action^106,107^ (e.g. thalamic connectivity to arousal structures is only reduced under propofol, not under dexmedetomidine,^99,102,103^ and the Lempel-Ziv complexity of spontaneous EEG in healthy volunteers is higher under ketamine than baseline at sub-anaesthetic dosages^104^). However, as consciousness is altered in all these conditions, common features affected by different neurotransmitters might help distil the NCC.^8^ Furthermore, unresponsiveness during anaesthesia does not necessarily imply the absence of mental content. Episodes of intraoperative awareness without explicit recall have been estimated to occur in up to 5% of the cases immediately after tracheal intubation^108^ and even more frequently in younger and female patients (up to 13%).^109^ Dreams can also occur during anaesthesia.^110^ Hence, despite similar behavioural states, there seems to be considerable variability which is thus far poorly characterized. Scientific work is underway in that direction.^110,111^

The application of anaesthetics in preclinical settings can also be relevant.^112-117^ In non-human primates, anaesthesia led to the attenuation of high frequencies and to a decreased spiking activity, paired with synchronized slow activity, putatively disrupting global dynamics, similarly to humans.^115^ For example, after the use of anaesthetic drugs, decoupling from anatomical networks has been reported in macaques,^118^ along with reduced interhemispheric cortical functional connectivity in mice,^102^ and different whole-brain connectivity patterns in rats.^99^*In vivo* rat experiments have identified that focal bursts of activity propagate throughout the brain orchestrated by the thalamocortical loop.^119^ These similarities suggest that animal studies under anaesthesia may serve as a preclinical testing ground for the assessment of treatment effects.

### How computational models can aid the identification of brain states

The development of computational models can help in understanding the dynamics of different brain states. For example, EEG data can be modelled using neural field models that allow fitting the power spectrum (*I2*) distribution by tuning thalamocortical connectivity and regional properties (*I3*), which can differentiate between levels of consciousness in DoC and sleep.^120^ Networks of coupled dynamical equations describing the activity of different brain regions have been used to get insight into the mechanisms underlying different brain states. These models are built considering the long-range white matter fibres connecting different brain regions and impose some hypothesized dynamics for the activity of the individual regions. Computational modelling is becoming a promising approach to the investigation of brain states of (un)consciousness. For example, models of sleep, anaesthesia and DoC have been employed to probe the effects of perturbations during those states, reflecting the empirical observation according to the level of consciousness.^121^ Previous studies in animal models under anaesthesia demonstrated that functional connectivity resembles structural connectivity more strongly, while during wakefulness activity tends to deviate from the structural backbone.^118^ Models have replicated this by showing that a decrease in the global strength of coupling leads to functional connectivity that looks closer to the structural connectivity as in awake, because the weak coupling only allows regions to interact with those with which they are directly connected, and interaction between regions that are two or three steps away becomes disrupted.^81,122^ Modelling work also highlights reduced network interactions,^81^ and reduced long-range connectivity in fronto-temporal regions.^122^ Modelling can also be effective at capturing the essential dynamics underlying sleep–wake transitions. This is also the case for the simulation of lesions and their effect on the brain-wide dynamics.^123,124^ However, given the heterogeneity in the oetiologies and brain lesions amongst patients with a DoC, it is challenging to make group-level predictions regarding brain regional involvement in consciousness. Nevertheless, in silico approaches can be used for strengthening insights in both capturing all features of a brain state (*I2*) and selecting those most relevant for supporting consciousness (*I3*).

### Induction of brain state changes

**Figure 4:**
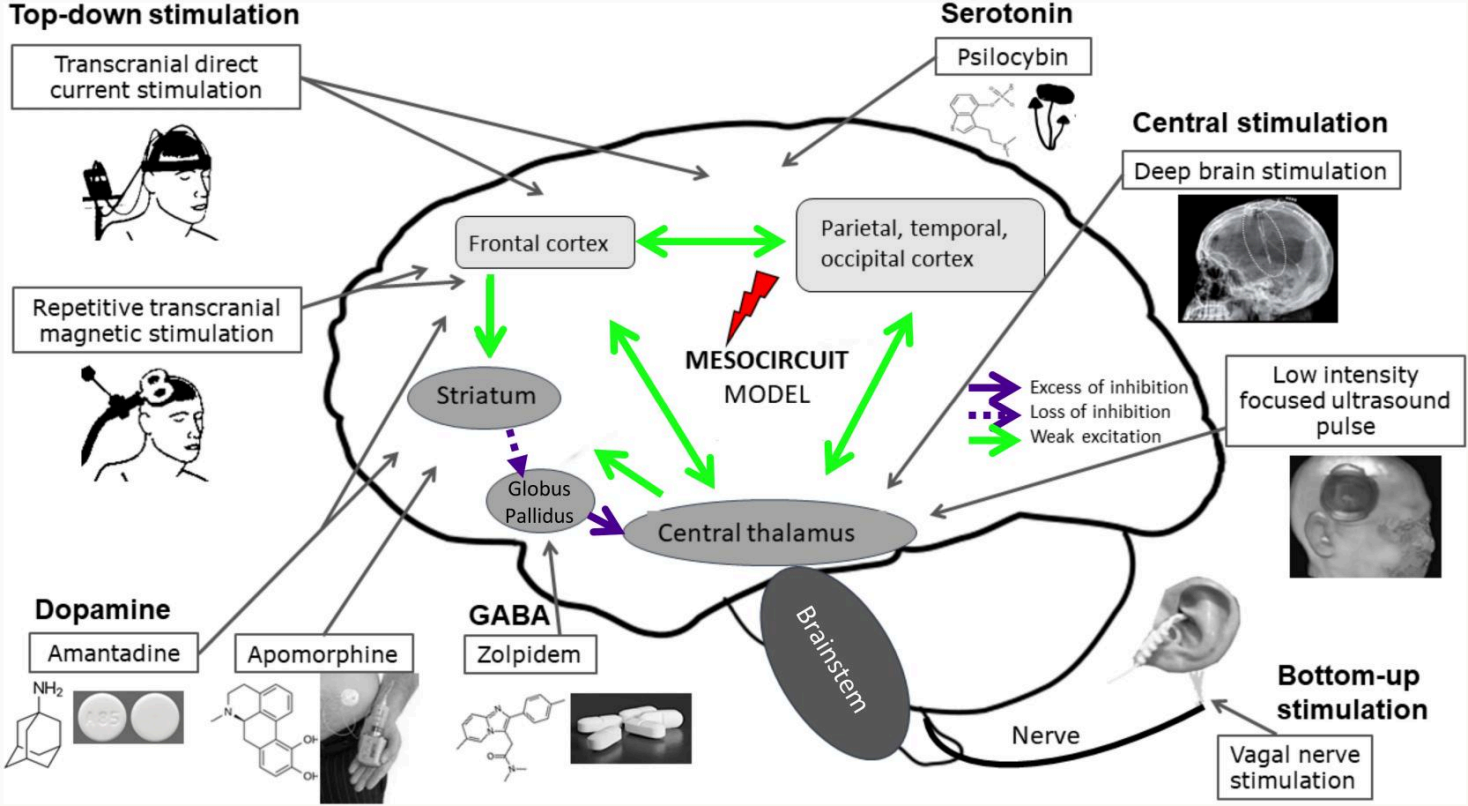
**Possible treatments tested for the therapy of patients with disorders of consciousness and their effect on the mesocircuit.** Pathways of weakened excitation (green, solid) and excessive (purple, solid; only between globus pallidus and central thalamus) or loss (purple, dashed) of inhibition that characterize patients with a DoC are shown in the mesocircuit model. From the top right, going clock-wise is shown: the serotonin system that is affected by psilocybin and acts cortically; central stimulation through deep brain stimulation (DBS) acts mostly on the thalamus; low-intensity focused ultrasound also affects the thalamus; vagal nerve stimulation stimulates the brainstem by nerve stimulation (latter three are bottom-up processes); the gamma-aminobutyric acid (GABA)ergic drug zolpidem targets the globus pallidus; dopaminergic drugs amantadine and apomorphine act on the striatum, while the former also affects the frontal cortex; repetitive transcranial magnetic stimulation (rTMS) and transcranial direct current stimulation (tDCS) act cortically and stimulate activity in a top-down fashion. Figure adapted from ^127^.

In healthy brain states, changes occur dynamically and relatively quick. This is evident in the different sleep stages, but also during wakefulness.^3^ Recovery of consciousness in patients with a DoC is a slow process that can occur at any time during a DoC. Recovery can occur spontaneously or be promoted by treatment. This induction of state changes can occur through pharmacology, photopharmacology, non-invasive brain stimulation, and deep brain stimulation. Some of these techniques are currently available and safe to treat patients with a DoC (*M1*). Even though few randomized controlled clinical trials have been performed in large samples, novel electrophysiological and pharmacological therapies have shown the potential to promote re-emergence of consciousness (reviewed in the study by Edlow *et al*.^125^). The state of the art of curative treatments for patients with DoC is often discussed in light of the mesocircuit model.^12^ In short, this model does not present itself as a theory of consciousness, but it describes cerebral malfunction in DoC as related to the widespread disruption of cortical neurones, causing a decrease in striatal activity due to the loss of thalamostriatal and corticostriatal connections. The reduction in striatal activity then inhibits thalamic function and leads to a decrease in both thalamocortical connectivity and activation (Fig. 4). To support this theory, the anticipated changes in the globus pallidus, striatum and frontal cortex measured with gamma-aminobutyric acid (GABA_A_) ligand precede functional recovery in patients with a TBI.^126^ Each of the presented treatments attempts to modulate the thalamocortical circuit. Identification of brain states and their most relevant properties could fit seamlessly to empirically drive the application of neuromodulation techniques for more effective treatment in DoC (*I > M*). The effect of treatment is often evaluated by assessing conscious behaviour (*M2*), and sometimes by measuring the effect on brain activity or brain states (*M3*).

### Pharmacological treatments for promoting consciousness in DoC

Neurotransmitters play a key role in the regulation of arousal and awareness and are therefore a good target not only to achieve unconsciousness (e.g. as often is the case through anaesthesia), but also to facilitate its recovery. Most pharmacological trials in patients with subacute-to-chronic DoC have tested stimulants that promote dopamine signalling, such as amantadine,^128^ methylphenidate^129^ and subcutaneous apomorphine.^130,131^ The most dramatic pharmacological improvement observed in patients with a DoC is achieved with the GABAergic agonist zolpidem. This sedative has shown a paradoxical arousing effect in some patients with DoC, but this effect is rare (∼5%^132^; *M2*^133^). This drug is thought to act on the globus pallidus interna, reducing its inhibitory effect on the thalamus. However, up to date, the only therapy that is supported by class II evidence in a randomized placebo-controlled trial is amantadine.^125,128,134,135^ The mechanism behind its association with accelerated recovery is still unclear, yet it appears to act as both a N-methyl-D-aspartate (NMDA) glutamate receptor subtype antagonist, and indirect dopamine agonist.^128^ Based on the mesocircuit hypothesis, dopamine could regulate the activity of the striatum to the globus pallidus, which, in turn, would reduce the inhibition of the thalamus and promote the activity of the frontal cortex, thus acting on the fronto-striatal-thalamic loop.^12^ Other pharmacological therapies that promote consciousness and neuronal function have been investigated with varying results,^50^ while others (e.g. levodopa, bromocriptine, modafinil, ketamine, selegiline and baclofen) are currently being explored.^136^ An ongoing clinical trial is currently investigating ketamine as a potential DoC treatment based on its ability to augment brain complexity at low/moderate doses. Theories linking the complexity of a brain state to consciousness suggest that the resulting transient augmentation of this complexity may improve the state of consciousness of patients with DoC (*M2*). Despite these modest successes, the gap between brain states and pharmacological treatments has not received sufficient attention (*M3*). This could be due to the challenge of understanding how the microscale mechanisms of action of these drugs affect the global dynamics.

### Photopharmacology as a tool to modulate brain states

**Figure 5:**
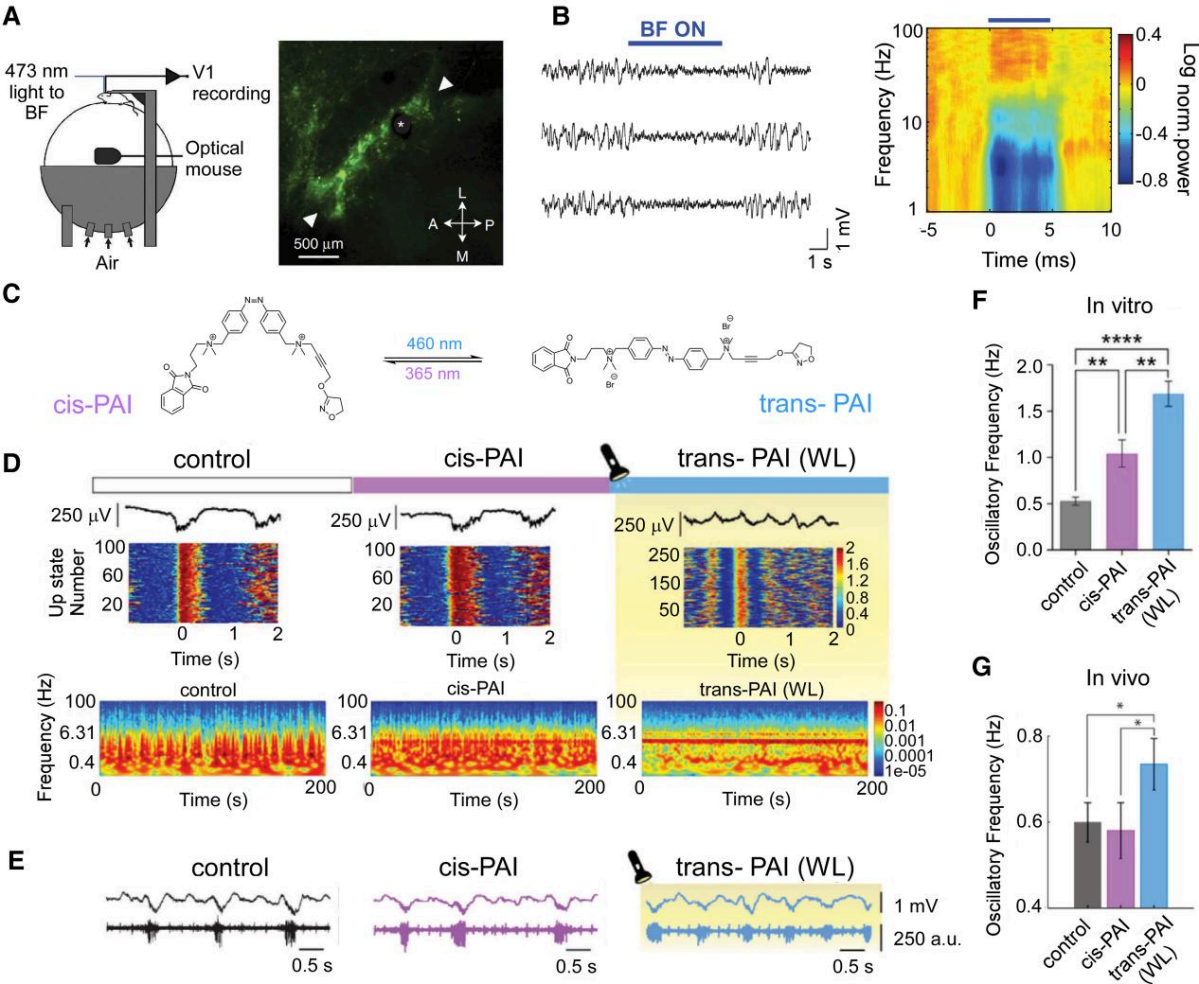
**Pharmacological neuromodulation-induced consciousness state changes in animal models. (A, B)** Optogenetic activation of basal forebrain (BF) cholinergic neurones desynchronize cortical activity in awake mice. Light activation of basal forebrain cholinergic neurones reliably desynchronized cortical activity by reducing the power at low frequencies (1–5 Hz) and increasing the power at high frequencies (60–100 Hz). **(A)** Schematic illustration of experimental setup (left) and fluorescence microscopy image of basal forebrain cholinergic cells expressing channelrhodopsin-2 and enhanced yellow fluorescent protein. Asterisk indicates the position of optic fibre; arrowheads indicate the posterolateral and anteromedial borders of basal forebrain **(B)** Three example local field potential (LFP) traces show the effect of basal forebrain stimulation (blue bar; average of all experiments in the right panel). Figure modified from the study by Pinto *et al*.^140^**(C–F)** Control of brain state transitions with a photo-switchable muscarinic agonist (Phthalimide-Azo-Iper (PAI)) *in vitro* and *in vivo*, and the effect on the brain network is studied. Physiological synchronous emergent cortical activity consisting of slow oscillations is transformed into a higher frequency pattern in the cerebral cortex, both *in vitro* and *in vivo*, as a consequence of PAI activation with white light (WL). **(C)** Chemical structures of trans- and cis-PAI photoisomers are shown. The molecule changes with light its capacity to bind to and/or activate proteins associated with neural signalling, reversibly switching from the straight trans configuration in the dark or under visible light, to the bent cis configuration when UV light is applied. **(D)** Photocontrol of brain waves *in vitro* using PAI and direct illumination with white light. Representative LFP traces (top), raster plots of firing rate during the Up-states (middle) and spectrograms (bottom) under control conditions, *cis*-PAI and *trans*-PAI after photoconversion with white light (WL). Colour scales are in arbitrary units (a.u.). **(E)***In vivo* photomodulation of brain waves. Representative raw traces of LFP (top, in millivolt (mV)) and multiunit activity (bottom, in arbitrary units (a.u.)), showing the differences in oscillatory frequency and firing rate during the Up-states between the control, *cis*-PAI, and *trans*-PAI after photoswitching with WL. **(F, G)** Changes in oscillatory frequency *in vitro* (**F**; mean ± SEM are reported from *n* = 17 ferret slices, one-way ANOVAs, with Welch test for pairwise comparisons, ** = *P* < 0.01, *** = *P* < 0.001) and *in vivo***G**; mean ± SEM are reported from *n* = 8 mice, one-way repeated-measures ANOVAs, with Fisher’s LSD for pairwise comparisons, * = *P* < 0.05) by PAI photoisomerization. Comparison of the different conditions analysed in this study: control, *cis*-PAI and *trans*-PAI. Figures C–F have been adapted from the study Barbero-Castillo *et al*.^152^ Access to interactive figures that allow for deeper investigation into the concepts illustrated here can be found in the Supplementary material.

The development of pharmacological treatments can be aided through photopharmacology. This can increase the spatial and temporal specificity of drug-based approaches, potentially increasing the control over the desired brain state. Various photo-switchable drugs have been developed and their deployment has allowed modulating brain states. Although only tested in humans for vision restoration (NCT05282953), localized drug administration has the potential to dramatically improve the efficacy and safety of diverse treatments. Exogenous neurotransmitters can be problematic at the systemic level, thus, localized drug action has been pursued by photopharmacology.^137,138^ Neuronal activity can be controlled with light by (opto)genetic expression of photo-switchable microbial proteins.^139^ Optogenetics has been used to transition the brain to a state of arousal both in awake and asleep rodents^140,141^ (Fig. 5A-B), yet its clinical translation to humans is hampered by the need for gene manipulation. This problem and potential immuno-reactivity are overcome by photopharmacology, which uses synthetic light-sensitive drugs targeting endogenous proteins.^142^ In short, the molecules can first bind to specific receptors or proteins involved in neural signalling and, by changing their configuration in the presence of light (Fig. 5C), alter receptor activity and subsequently neural behaviour to adhere to desired patterns. According to the mesocircuit hypothesis, it is conceivable to mitigate the neuronal damage in patients with a DoC with either a photo-switchable antagonist molecule binding to GABA receptors or an agonist binding to glutamate receptors. The distribution of this drug can be throughout the brain, but the action of these drugs could selectively be controlled with precise timing, for instance by focused light stimulation in the globus pallidus (Fig. 4). Several photo-switchable inhibitory ligands have been studied, including derivatives of the anaesthetic propofol,^143,144^ fomocaine,^145^ and glycine receptors^146^ potassium channels^147^ and the mild sedative clonidine (termed adreno-switches).^148^ The potentiation of GABA_A_ receptors with light has also been demonstrated using photouncaged diazepam,^149^ benzodiazepines^150^ and tethered propofol derivatives.^151^ Localized GABA_A_ administration to the globus pallidum might decrease its inhibitory effect on the thalamus,^12^ while avoiding the depressing effect of GABA_A_ on the rest of the brain. All these compounds display pharmacological profiles that offer the potential to control arousal with light in mammals, as recently demonstrated using a photo-switchable muscarinic agonist that controls cholinergic-dependent brain state transitions in anesthetized mice^152,153^ (Fig. 5C-G). Synchronous emergent cortical activity, similar to slow-wave sleep, was transformed into a higher frequency pattern both *in vitro* and *in vivo*, by activation of a muscarinic agonist with light. These results pave the way to study neuromodulation by cholinergic ligands (including recently developed photo-switchable antagonists^154^). Together, they offer the promise of controlling spatiotemporal patterns of activity in different brain states and facilitate their transitions to wake-like patterns.

Animal studies also provide possibilities to test specific hypotheses regarding the role of specific brain regions in sustaining brain states of reduced or normal consciousness. This can be done by performing lesion studies, but also by the application of (local) (photo)pharmacology or brain stimulation, in an attempt to activate brain regions (*M3*). Alternatively, photosensitive muscarinic receptors could be used to functionally and transiently ‘lesion’ one single brain region at a time *in vivo*. Such an approach would allow investigating of how the thalamocortical loops and specific resting-state networks influence whole-brain dynamics and brain states.

Thus, photopharmacology is a promising tool to achieve high spatiotemporal control of drug actions (Fig. 5) without genetic manipulation. Techniques like near-infra-red spectroscopy (NIRS)^155^ and photobiomodulation allow sending light pulses into the human brain non-invasively.^156^ Advances in the use of red^157^ and pulsed infra-red light (two- and three-photon excitation^158,159^) make photopharmacology compatible with transcranial non-invasive illumination that, in the long term, could be used in humans. Yet, the clinical translation of animal research is unsure and will require careful investigation, but these preclinical trials allow the development of promising tools for the promotion of consciousness-associated brain states.

### Non-invasive brain stimulation for promoting consciousness treatment of DoC

Electromagnetic stimulation techniques are established in clinical practice for the treatment of specific diseases such as major depressive disorder.^160^ Research in the past decades has explored several non-invasive brain stimulation techniques as therapeutic options for promoting consciousness in patients with DoC. Brain activity can be stimulated with repetitive transcranial magnetic stimulation (rTMS) which could promote consciousness recovery by top-down stimulation of the cortex that can increase neuronal excitability of specific brain regions. One study stimulating the left primary motor cortex showed increases in cerebral blood flow in MCS, but not in UWS patients.^161^ rTMS applied over the motor cortex has shown little behavioural effects (*M2*).^162-164^ However, more recently, the effects of rTMS seem stronger when targeting the left prefrontal cortex.^165-167^ Stimulation over the left parietal cortex has also shown promising improvements in behavioural scores in MCS^168^ and even UWS patients.^169^ Interestingly, rTMS has been shown to increase levels of the oestradiol hormone in responders,^166^ which in turn has been shown to be capable of influencing brain states by restoring interhemispheric balance.^170^ Together, these preliminary studies collectively suggest that rTMS is a valid and safe treatment option in DoC patients.

Among the non-invasive brain stimulation techniques, tDCS is currently one of the most popular and well-studied,^171,172^ as it is easy to apply and inexpensive. tDCS modulates membrane polarity and neuronal excitability, allowing for close-loop activity control^173^ and producing long-term depression and potentiation-like mechanisms. In a pivotal randomized placebo-controlled cross-over trial of tDCS over the left dorsolateral prefrontal cortex (DLPFC), as a therapeutic tool to enhance consciousness in patients with DoC, it was found that 43% of MCS patients (i.e. 13 out of 30) showed significant behavioural improvement (*M2*).^174^ This might be a result of increased functional connectivity from the site of tDCS stimulation (DLPFC) to frontal and parietal brain regions.^175^ In an effort to understand and reproduce this outcome, the associated brain states need to be investigated, along with a protocol and stimulation-site optimization (for more extensive discussion, see the study by Barra *et al*.^40^). Regarding targets of stimulation, apart from dlPFC, the precuneus,^176^ and primary somatosensory area^177^ have shown behavioural improvement, while the primary motor cortex^178^ or the fronto-parietal network^179^ were not associated with significant behavioural changes.

**Figure 6:**
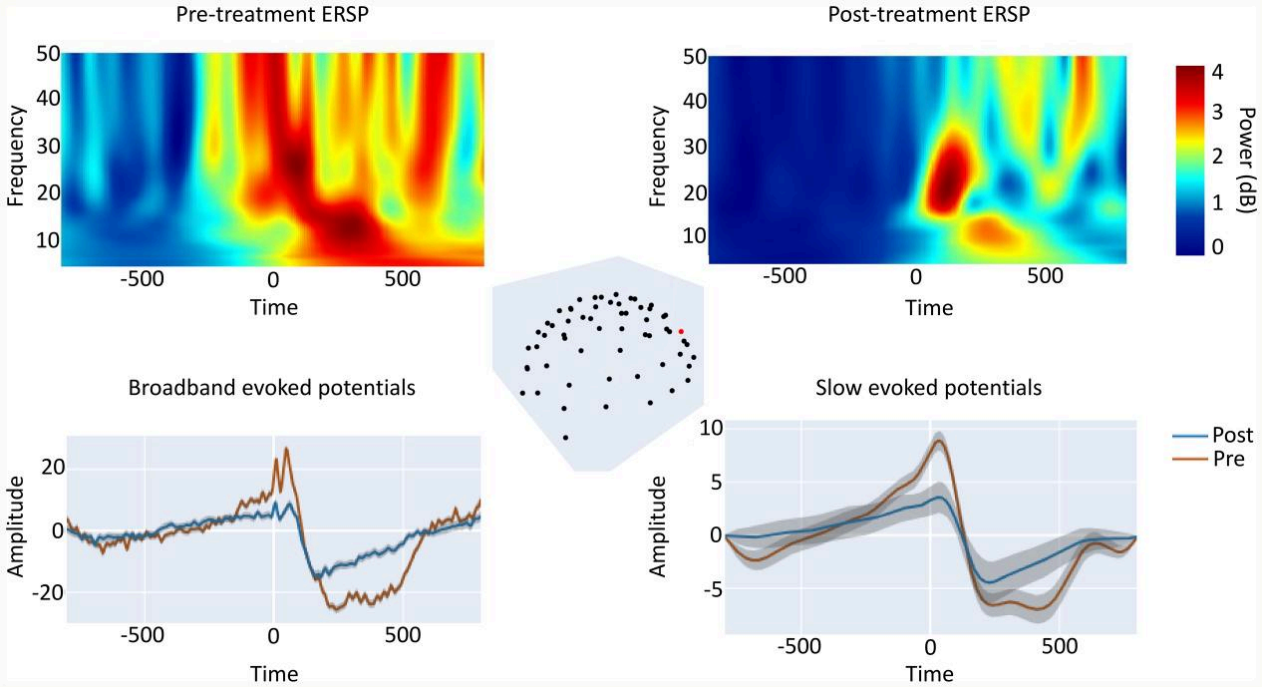
**Neurophysiological effects of tDCS over the dorsolateral prefrontal cortex in a patient in the minimally conscious state**. EEG data evoked by a transcranial magnetic stimulation (TMS; −800 ms before to 800 ms after, on each *x*-axis) before and after transcranial direct current stimulation (tDCS) treatment (see the study by Mensen *et al*.^4^ for experimental details). The top left figure shows the event-related spectral perturbation (ERSP; frequency on the *y*-axis, colour indicating increasing power blue->red) pre-treatment while the right figure shows the ERSP post-treatment. A marked decrease in the slow wave induced by the TMS pulse can be observed. The top middle figure displays the electrode configuration, with a red dot indicating the electrode for which the responses are displayed. The bottom figures show the pre-treatment (red) and post-treatment (blue) amplitude response (*y*-axis) for both the broadband and the filtered (2–6 Hz) signals on the left and right, respectively. Lines display mean responses over all TMS trials, shaded area shows standard deviation. These figures are from a single subject (*n* = 1) to illustrate that all individuals showed significant differences between pre- and post-tDCS treatment (statistics not reported, but clear from standard deviations). Despite the changes in brain state as a result of this tDCS treatment there seems to be an absence of behavioural effects. Access to interactive figures that allow for deeper investigation into the concepts illustrated here can be found in the Supplementary material.

Besides the stimulated area, more parameters seem to influence the effectiveness of tDCS. Patients who respond to stimulation show more preserved grey matter in areas that are considered critical for consciousness (e.g. precuneus and thalamus) and greater overall metabolism than non-responders.^180^ Responders at rest before tDCS were characterized by higher theta connectivity and network centrality compared to non-responders.^69^ Even in the absence of behavioural improvement, tDCS can cause changes in the brain states as evidenced by EEG.^181,182^ One study (Mensen *et al*.^4^; Fig. 6) explored the effects of tDCS on TMS-evoked potentials and found that tDCS significantly reduced the amount of slow-wave activity but did not produce an increase in high-frequency suppression. As the patients in this study did not show any behavioural improvement, it has been suggested that reduced slow-wave activity alone is not sufficient without an increase in higher frequencies. Alternatively, these findings might suggest that conscious brain states could be stimulated with tDCS, but that behaviour is limited by physical impairments, leading to the presence of covert consciousness.^5,29,183^ Latest multicentric studies highlight the modest effects of tDCS on consciousness, as significant treatment effects 3 months after 4 weeks of treatment were only observed in MCS patients who suffered a traumatic brain injury.^183^ Given its relative ease of use, limited costs, and potential future developments, tDCS remains an appealing option for the treatment of DoC patients.

Thus, non-invasive brain stimulation is considered an acceptable manner to modify the brain state (*M3*), sometimes coupled with behavioural improvements in patients with a DoC (*M2*). Interestingly, both for rTMS and tDCS, the top-down targeting of the prefrontal cortex seems to be the most promising area to stimulate. This is in line with the mesocircuit model, as the prefrontal cortex has direct projection to the striatum, which could in turn, promote the fronto-striatal-thalamic loop. More recently, small sample open-label studies have employed a variety of techniques to act on the brain in a bottom-up manner. Transcutaneous vagal nerve stimulation provides another promising outlook, as it can stimulate cerebral activity through the modulation of brainstem activity.^184^ In brief, vagal nerve stimulation, through its indirect connection to the locus coeruleus and the raphe nuclei could promote norepinephrine and serotonin release which act on specific brain regions, but most importantly on the thalamus.^185^ Also, the effect of ultrasound stimulation has been investigated,^186^ which can act directly on the thalamus to restore brain activity while being non-invasive.

### Deep brain stimulation for promoting consciousness in DoC

**Figure 7:**
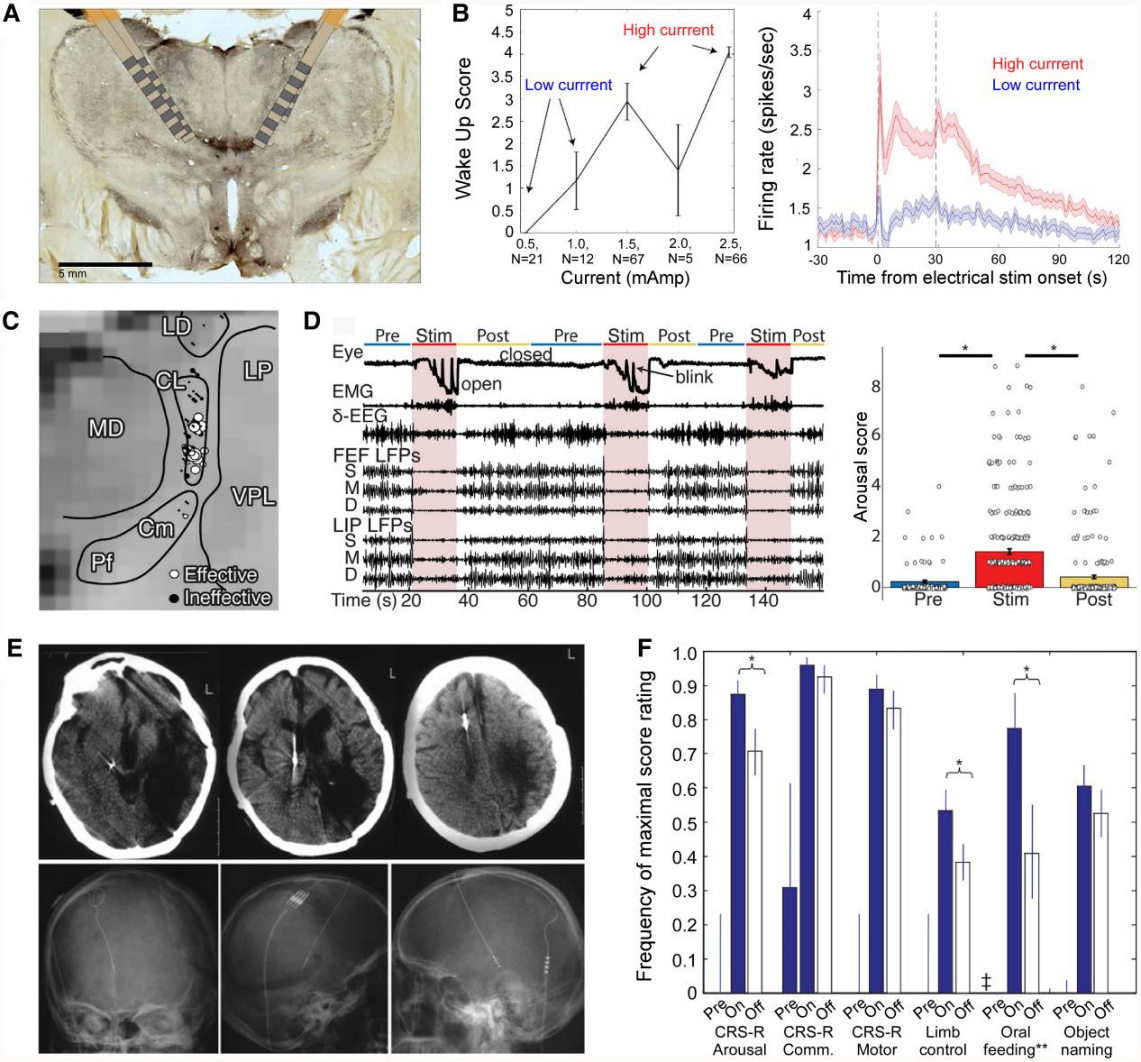
**Electrical-induced state changes to promote consciousness in animals and humans by means of thalamic stimulation.** Consciousness depends on large-scale thalamocortical and corticocortical interactions. Many studies support non-specific thalamic nuclei (intralaminar nuclei) as critical structures. **(A-B)** Thalamic electrical stimulation in central thalamus arouses monkeys (adapted from the study by Bastos *et al*.^115^). **(A)** The histological images, using an acetylcholinerase staining, show the thalamic stimulation leads in the central thalamus. **(B)** The effects of thalamic electrical stimulation on cortical state in monkeys are shown by an example of the behavioural wake-up score as a function of thalamic current (left) and the mean firing rates with respect to electrical stimulation onset and offset across all cortical areas (right).^115^**(C-D)** Central–lateral thalamic stimulation arouses macaques from stable anaesthesia (adapted from the study by Redinbaugh *et al*.^42^). **(C)** Stimulation sites (*n* = 90) in one subject collapsed along the anteroposterior axis are shown in the image. Circles represent the middle contact in the stimulation array, diameter scales with induced arousal. **(D)** An example of the behavioural and neural recordings during 50-Hz stimulation is shown in the left panel. The population mean arousal score pre, during (stim) and post stimulations is represented in the right panel. Using linear mixed effect models over all stimulations (*n* = 261) pre significantly differed from during the stimulation (*F* = 119.28, * = *P* < 0.001) and during stimulation significantly differed from post (*F* = 124.64, * = *P* < 0.001). Other abbreviations: MD, mediodorsal thalamic nuclei; LD, laterodorsal thalamic nuclei; LP, lateral posterior thalamic nuclei; VPL, ventral posterolateral thalamic nuclei; EMG, electromyography; FEF, right frontal eye field area; LIP, lateral intraparietal area; S, superficial layers; M, medium layers; D, deep layers. **(E–F)** The electrical stimulation of different intralaminar nuclei has been demonstrated to restore consciousness in patients with disorders of consciousness. **(E)** Example of deep brain stimulation (DBS) for treatment of a patient with the unresponsive wakefulness syndrome. The stimulating electrode was implanted for stimulation of the CM-PF. Computerized tomography (upper) and radiography (lower) show the trajectory and location of DBS electrode (adapted from the study by Yamamoto *et al*.^203^). **(F)** Bilateral DBS of the central thalamus modulates behavioural responsiveness in a patient who remained in minimally conscious state for 6 years following traumatic brain injury before the intervention. Comparison of pre-surgical baselines of achieving the maximal obtained behavioural score with this same metric with DBS on and DBS off periods during the cross-over phase (adapted from the study by Schiff *et al*.^195^). Evaluated using two-tailed Pearson chi-square tests, where * = *P* < 0.001, Significant differences can be found between DBS-on and DBS-off for CRS-R arousal scores as well as limb control and oral feeding, all of which are better with DBS on.

While non-invasive techniques are generally less risky and easier to try, invasive neuromodulation techniques can reach deeper structures with more precision and could induce stronger beneficial effects. The thalamus is not a single entity but consists of a great number of sub-nuclei. Inside the medial medullary lamina are the intralaminar nuclei, with, importantly in the caudal part, the centromedian parafascicular nuclei complex (CM-PF) that has glutamatergic afferents to the striatum beside some output to the nucleus accumbens, other parts of the basal ganglia, midbrain and cortex^187,188^ in a diffuse way (reviewed in the study by Clasca^189^). These diffuse projections allow the nuclei to influence the overall excitability of the cortex and are implicated in consciousness^190^(see below, Fig. 7). On the other hand, a small lesion in the intralaminar thalamic nuclei can cause loss of consciousness.^191^ Furthermore, studies with patients with a DoC have reported reductions in functional connectivity restricted to the cortico-thalamocortical networks from the intralaminar nuclei^192^ The DBS of different intralaminar nuclei and adjacent portions of the mediodorsal, ventral lateral and anterior pulvinar nuclei has been demonstrated to ‘awaken’ anesthetized non-human primates and reverse electrophysiological features of unconsciousness, restoring both the signatures of arousal and awareness (^42,115,193^; Fig. 7A-D). In addition, DBS of this ‘central thalamus’ has been used successfully, mostly in case studies, to restore cognitive functions and increase consciousness levels in DoC (Fig. 7E and F).^194-197^ It has also been shown to be effective in facilitating memory and attention in rats.^198^ This heterogeneous collection of nuclei together innervates the dorsolateral prefrontal, premotor, posterior parietal and cingulate cortices and the dorsal striatum, which are key nodes of the brain’s attention, executive control, and working-memory networks.^199^ Clinical DBS in the thalamus focuses on the central–lateral (CL) nucleus, to restore arousal regulation sufficiently to support communication or to restore executive cognitive function.^195,200,201^ Recent studies in rodents and non-human primates have shown that the electrical activation of the central thalamus can either drive the brain to an ‘awake’ state or promote a state of unconsciousness, depending on the parameters of the stimulation (*M3*).^141,202^ Another study provided evidence that the CL nucleus supports consciousness through modulation of neocortical intra-columnar and inter-regional interactions in macaques,^42^ specifically showing an enhancement of corticocortical synchrony in the gamma range.

Apart from the thalamus, other studies in rodents demonstrated recovery from anaesthesia induced by site-specific electrical stimulation in different subcortical structures that are part of the AAN distributed across the brainstem.^204^ Stimulation of the parabrachial nucleus of the pons has been proposed to regulate arousal^205^ and has been shown to cause awakening from anaesthesia (*M2*).^206^ In parallel, the stimulation decreases EEG delta oscillations (*M3*). Electrical stimulation of the pontine reticular nucleus also decreases delta and theta power under anaesthesia and increases the integration of cortical information, both spontaneous and stimulus-evoked.^207^ Finally, stimulating the ventral tegmental area, a main source of dopamine in the brain, increased responsiveness^208^ and was accompanied by a shift in peak frequency from delta to theta range.^209^

With increased precision at the cost of invasiveness, DBS, especially to nuclei of the thalamus but also to parts of the AAN, has been successful in manipulating brain states (*M3*) and even inciting recovery from anaesthetically induced unconsciousness (*M2*). In most of the fruitful approaches, brain state changes followed the same divergence between conscious and unconscious states that are described above in the investigation of brain state identification: reducing slow oscillations and increasing faster ones, as well as increasing functional connectivity patterns and complexity.

### Computational modelling approaches to induce brain state changes

While empirical studies show promising avenues for the development of treatments that can promote or induce brain state changes and/or cognitive/behavioural changes, they are limited by a lack of data on individual brain injuries of patients. Computational modelling approaches provide the possibility of adapting the model for each patient, opening the door to personalized investigations.^210^ Such an approach (*M3*) is particularly promising, given that each DoC patient’s lesion is unique. While this individualized approach is still in its infancy, it could result in major steps towards successful treatment of patients with a DoC.^211^

Another factor that limits empirical treatment research pertains to the focus of the treatment. In the case of pharmacology, the treatment targets the whole brain. Photopharmacology, targeting specific regions, provides a better solution; however, its clinical application is not yet accessible. Electrical stimulation can reach a specific region of interest; however, the frequency and duration are unknown, and optimal settings are being explored empirically. Fine-tuning these parameters requires many randomized controlled trials, which are challenging. Another approach could be perturbing the model to result in a state change from sleep to wakefulness and back (*M > I Confirmation*),^2^ serving as a promising building ground for the modelling of state transitions in patients with a DoC. Such an approach saves time and allows us to test the effect of large changes (e.g. stimulation location), but also allows us to fine-tune parameters (e.g. small changes in stimulation frequency) to achieve the best possible results. In line with a recent review, it seems that computational modelling is a reliable and robust way to characterize brain states and induce changes regardless of their context.^212^ Modelling has already been used to show that sleep–wake transitions across 17 animal species share a common physiological background.^213^ Models without hypothesized structure or dynamics, such as maximum entropy models, can distinguish between awake and anesthetized states and quantify the system’s capabilities for information processing.^214^ Such data-driven approaches might be powerful in the context of DoC, where hypothesized parameters might be unreliable due to brain injury, and where the ground truth of the patients’ state is lacking. Consistently adding modelling approaches to a common toolbox of professionals interested in consciousness can be implemented by creating adherence of generative models to rules derived from theories of consciousness that lean on their empirical observations.^215^ Elegant computational measures that quantify regional in- and outflow after simulated perturbation can help uncover minimal conditions that allow for the recovery of (partial) consciousness, as demonstrated in MCS patients who have been characterized to integrate information better in a posterior network and broadcast information better in the fronto-temporal-parietal network.^216^ Data-constrained computational brain models have also contributed to explore neurobiological mechanisms of conscious perception, for example, the potential origin of ignition thresholds in prefrontal areas,^217,218^ and they constitute a promising tool to approach DoC in this context. However, concrete predictions on how state changes could be induced (i.e. which settings and techniques) to help patients improve their consciousness are still not possible.

### How the framework can help unite brain states identification with neuromodulation

Despite the vast number of recent advances and sometimes important successes in the fields of brain state research of consciousness and the neuromodulation of consciousness, mutual benefits could be achieved with more direct interactions between these fields. Regarding the extraction of brain states (*I2*), it becomes apparent that common features can be identified, which might be universal to being conscious, regardless of the causes and specific conscious content. However, although from an outsider’s perspective, various different ways of losing consciousness may appear similar, it has been argued that the mental state of sleep and anaesthesia are different.^219^ In this sense, we should be cautious in aggregating data across multiple domains and a concerted effort should aim to conduct comparative research across different states of (un)consciousness. For example, anaesthetics have been shown to alter neurovascular coupling, complicating the interpretation of how these changes might be related to brain states and consciousness specifically.^220^ Despite these differences, the onset of consciousness (or maintaining a conscious state) requires some minimal conditions to happen. Thus, although anaesthesia, sleep, and DoC might follow different mechanisms, their common phenomenology across reasons should make it easier to identify the minimal conditions for brain states (not their causes) to support consciousness. The differentiated causes can be simulated via computational modelling. The same model of brain activity can be perturbed in different ways (different mechanisms) to display the ‘same’ unconscious-like dynamics. To further the current understanding of the dynamic brain state, different levels of analysis must be integrated to capitalize on the complementary insights they offer. It remains to be seen how combined multimodal data (e.g. EEG and fMRI), acquired during the same physiological and behavioural brain state, can be used to improve our understanding of their relation. Before large-scale clinical translation is likely to occur, it is important to investigate which modality and extracted feature collection is most accurate and relevant (*I3*) in the identification of brain states in the DoC population. Not only the sensitivity and specificity of each technique is relevant. More practically, EEG at the bedside may be more feasible on a large scale^64^ than fMRI. EEG availability is widespread and, because of the relative insensitivity to the number of trials and electrodes, large samples collected across multiple sites could serve as benchmark.^64^ Benefits like this need to be weighed against the potential for accurately capturing the relevant features (*I3*) of the brain state under investigation.

Also animal models can provide unique novel insight by means of e.g. lesion studies. The precise experimental control that can be achieved with animal models and the use of anaesthesia could pave the way for future clinical and fundamental knowledge, given broad similarities in their findings to human research. Such development could aid the confirmation of consciousness and the significance of consciousness-supporting brain states (*M > I*), specifically by mapping the critical points on the trajectory from unconsciousness to consciousness and stratifying individual DoC patients on the continuum. This can be paralleled by the development of computational modelling and statistical approaches that allow for the proper extraction of relevant brain state features. The statistical approaches should aim for generalization (e.g. using cross-validation in a procedure to extract the most relevant features to discriminate UWS and MCS^64^; Fig. 3B), be robust and able to bridge across datasets,^221^ as is done for the decoding of cognitive tasks from functional neuroimaging data.^222^ Additionally, self-supervised learning approaches could aid in identifying relevant groups to extract features from, as has been done in sleep research.^223^ In the end, the balance between experimental control and clinical relevance, tied with computational approaches, should be integrated to cover the entire proposed framework.

Crucially in this review, we see that treatment options to increase consciousness in patients with a DoC target aspects of the brain states associated with unconsciousness (e.g. tDCS to the prefrontal cortex to normalize DMN connectivity,^224^ or DBS in the thalamus to increase cortical function^194-197^). Still, a barrier to developing consciousness-promoting therapies has been the lack of target biomarkers, such as the precise identification and characterization of brain states, which would allow a more objective assessment of the therapeutic responses.^125^ We believe that tracking brain states more systematically would allow the refinement of treatment protocols, and ultimately increase behavioural effectiveness. With a better characterization of brain states associated with consciousness, sub-clinical changes following an intervention could be captured, which would improve not only treatment efficacy but also covert consciousness diagnoses. Likewise, the development of closed-loop neuromodulation systems^225^ could improve treatment efficacy (*M2*) by stimulation during specific brain states (*I3*). Treatment might be more efficient in specific brain states, and a preliminary investigation in this direction is ongoing in the form of closed-loop tDCS monitoring arousal and stimulating in high- or low-arousal states specifically.^226^ In this way, the fields of brain state identification can be beneficial to the induction of brain state transitions.

With regard to the investigation of state transitions in patients with a DoC, behavioural treatment responses (*M2*) are often relatively small in comparison to, for instance, the paradoxical but dramatic changes following zolpidem. Moreover, responders to any treatment are mostly MCS patients, whereas patients in UWS do not often show significant improvement. This poses the question if, up to now, it had not yet been possible to induce brain state changes, or if some patients’ curative treatment would be forever futile. The heterogeneity observed amongst patients will skew research towards more personalized protocols. Montages and stimulation settings are varied and far from being exhaustively explored. Furthermore, other personal and contextual factors such as the vigilance state of the patient at the moment of stimulation could be a crucial factor, as in the closed-loop system example.^226^ Such a closed-loop approach could simultaneously stimulate but also measure the effect on ongoing dynamics and brain states (*M3*). Other factors to consider for the positive outcome of the treatment could be the present resting-state dynamics, as becomes apparent from the identification of responders or the subjects’ brain lesions. Certainly, if the stimulation electrode is placed on a skull area that is above a brain lesion, the stimulation might not be effectively delivered.^227^ Furthermore, animal models could help in defining treatment protocols. However, currently, different protocols of DBS in human and animal analogues (e.g. stimulation intensity differing in magnitude)^228^ are being used which might result in conflicting results in human and animal research on the mechanisms of treatments (e.g. DBS^229^). Computational models to simulate the effects of specific treatments might provide a way forward. Ideally, they should be developed at the single subject level, accounting for specific lesions, to tune and estimate the desired effects in silico. This could improve treatment, but also open doors to the use of more experimental treatment options including the use of psychedelics.^211^ In this way, computational modelling could act as a drug discovery tool facilitating ‘phase-zero’ clinical trials which circumvent potential ethical problems and provide initial support for or against theoretical predictions.^230^

Thus, the field of neuromodulation can help the identification of brain states for consciousness by empirically confirming their relevance (*M > I confirmation*), complementary to the identification of brain states informing the target (*I > M*). This mutual benefit is unfortunately not clearly illustrated in the present literature. The most fitting approach would be the ongoing closed-loop protocol where *I3* provided the targeted brain state feature of entropy, informing *M3*, where consequent measurements should see if the neuromodulation increases the brain state entropy, confirming its relevance. Going further, it would be of interest to not only validate the relevance of specific brain state dynamics, but also to investigate if the target brain states were the most relevant to consciousness, or if other brain state dynamics were more strongly related to cognition/behaviour.

The framework is inclusive towards many types of research and actually fosters cross-discipline collaborations. However, for example, animals and *in vitro* studies, there would be a long way to the actual clinical implementations of these techniques. The development of new neuromodulation tools in animal models and their translation to clinical reality also emphasizes the need to perform extensive efficacy and safety studies, as well as exhaustive evaluation of ethical concerns related to it (e.g. when and how can we utilize the proposed new technique in humans? ). Furthermore, the focus of the current review was mostly on global brain states and associated behaviours. However, brain states can have distinctive local characteristics seemingly out of tune with the behavioural state. For example, during local sleep, where only parts of the brain can display distinct sleep-like electrophysiological patterns, all in a behavioural state of wakefulness.^231^ On the contrary, hypothetical islands of awareness in certain brain regions would allow preserved awareness in a behavioural state of unconsciousness.^232^ As such, approaches to the identification of brain states can focus on the whole brain or single-out local dynamic patterns. Even slice preparations or cells could be included in the framework to study specific aspects or facilitators for consciousness-related global brain states. For example, it was shown that *in vitro* experimentation of mice brain slices decreased bursts of neuronal spikes and spreads under isoflurane anaesthesia.^233^ Such fine-grained assessment of neuromodulation can, alongside multimodal whole-brain assessments, help to develop new neuromodulation techniques.

## Conclusions

A large body of research shows that crucial features of conscious brain states are lost during unconsciousness. The conscious wakefulness is characterized by higher frequencies in the EEG and by a richer dynamical repertoire of resting-state networks in functional connectivity. Overall, complex brain states seem to reliably indicate consciousness. The developments in the identification of brain states make it now possible to go beyond exploratory approaches and embrace an open science framework of confirmatory testing. Promoting these consciousness-associated brain states through various specific targets is the main avenue for neuromodulation, which paves the way to improvements in the level of consciousness of patients with a DoC. This process could start by identifying a target brain state, followed by the formulation of a hypothesis regarding the procedures that may induce this state. Next, choosing the appropriate methodology should be done along with pre-registering these plans prior to conducting the experiment. Computational modelling and individualized approaches should be taken into account as they can pave the way towards better patient care. By matching both the brain state and neuromodulation fields, we believe that it is now possible to further develop rigorous, theory-driven, large-scale confirmatory research to end up with a fundamental understanding of consciousness, its alteration, and associated clinical conditions. The proposed therapeutic framework for identification and modulation of brain states has the potential to propel the field on a path of greater maturity.

## Supplementary Material

fcae362_Supplementary_Data

## Data Availability

No new data were created for this study, but data created for the previous studies that form the basis of the figures are shared through EBRAINS, alongside interactive figures to explore this data further. For data, see Fig. 1 (t.ly/edVHU), Fig. 3 (t.ly/xjoSy), Fig. 5 (t.ly/qSE8b) and Fig. 6 (t.ly/kqi9M).
