# Supplementary material for: Brain state identification and neuromodulation to promote recovery of consciousness
Source: Brain Commun. 2024 Oct 11;6(5):fcae362. doi: 10.1093/braincomms/fcae362 (PMC11520929; doi:10.1093/braincomms/fcae362)
Supplement: fcae362_Supplementary_Data [file fcae362_supplementary_data.docx]

**Supplementary Material**

**Interactive figures**

This paper presents interactive figures, supported by the Live Paper initiative of the Human Brain Project, enabling the interaction with data and figures illustrating the concepts in the paper through EBRAINS (go to <https://wiki.ebrains.eu/bin/view/Collabs/live-paper-states-altered-consciousness> and get started with an EBRAINS account). Below the links for each interactive figure are shared. For all interactive figures an EBRAINS account is required. When clicking the link: Click “Sign in with GenericOAuth2” and proceed to login to EBRAINS. It may be required to click the link again after logging in.

**Figure 1. The arousal-awareness axes of consciousness and examples of metabolic and functional connectivity in patients with disorders of consciousness.** Go to EBRAINS ([t.ly/edVHU](https://t.ly/edVHU)) to view the live version of this figure. The live version of the figure contains 3 buttons ("Healthy", "MCS", "UWS") to select different states of consciousness. Panels B (FDG-PET) and C (EEG) will automatically be updated, showing example data from subjects in these states. The live version of the figure contains a slider that allows to scroll through transversal slices of the PET and allows rotation of the EEG 3D figure and inspection of all sides.

**Figure 3. Methods for identification and characterization of brain state dynamics illustrated through fMRI connectivity patterns.** Go to EBRAINS ([t.ly/xjoSy](https://t.ly/xjoSy)) to view the live version of this figure. The live version of this figure allows the user to vary this thresholding percentage dynamically, rotate the brain in 3D and to view the name of every brain area upon hovering to explore these brain states in more detail.

**Figure 5.** **Pharmacological neuromodulation induced consciousness state changes in animal models.** Go to EBRAINS ([t.ly/qSE8b](https://t.ly/qSE8b)) to view the live version of this figure. The live version of this figure allows the user to inspect the LFP (Local Field Potential) and MUA (Multi-Unit Activity) traces for 3 different brain slices and for control, *cis* and *trans* conditions. The user can then select the channel (based on the electrode location provided) and visualize how the frequency of the slow oscillation changes along with the corresponding rastergrams.

**Figure 6. Neurophysiological effects of tDCS over the dorsolateral prefrontal cortex in a patient in the minimally conscious state**. Go to EBRAINS ([t.ly/kqi9M](https://t.ly/kqi9M)) to view the live version of this figure. In the live version of this figure, a dropdown menu allows you to select different electrodes and view their responses. Electrode configuration is rotatable and electrode names are displayed upon hovering over them.
